# The complete mitochondrial genome of an ornamental firefly *Pyrocoelia analis* (Coleoptera, Lampyridae)

**DOI:** 10.1080/23802359.2022.2078239

**Published:** 2022-06-01

**Authors:** Xiaoyang Ji, Huachao Xu

**Affiliations:** Department of Forestry Protection, School of Forestry and Biotechnology, Zhejiang A&F University, Hangzhou, China

**Keywords:** Mitogenome, glowworm, ornamental insects

## Abstract

*Pyrocoelia analis* (Fabricius, 1801) (Coleoptera, Lampyridae, Pyrocoelia) is a beautiful ornamental insect widely distributed in East and Southeast Asia. The complete mitogenome of *P. analis* has been sequenced. The mitogenome, total length of 14,785 bp, includes 13 protein-coding genes, 22 transfer RNAs, two ribosomal RNAs, and a noncoding D-loop region. The overall base composition of *Pyrocoelia analis* mitogenome is 34.63% for A, 13.69% for C, 42.79% for T, and 8.89% for G, with a high A + T bias of 77.42%. These mitogenome data might be useful for further phylogeography analyses and other related studies in Hymenoptera.

*Pyrocoelia analis* (Coleoptera, Lampyridae, Pyrocoelia), a beautiful and popular ornamental insect in China, is widely distributed in East and Southeast Asia (Osozawa et al. 2015). The body length is 14–21 mm, which is larger than glowworms, and it is like an elf in the forest, it has more ornamental value than ordinary fireflies. *Pyrocoelia analis* in the larval stage also has great ornamental value. But in the larval stage, *Pyrocoelia analis* is difficult to distinguish from other glowworms of *Pyrocoelia*, then mitochondrial genome sequencing is needed to help distinguish the larval classification (Gill et al. 2003; Jeng et al. 2011). Previous studies on this species mainly have been focused on taxonomic description and biological habit of adults, and only a few studies have been done on genes. However, genetic characteristic is the key factor in the study of biodiversity and phylogeny, so elucidating the mitochondrial genome structure of *Pyrocoelia analis* is crucial to understanding its diversity and evolution (Zhao et al. 2019).

The insect material of Pyrocoelia analisI was collected from Wuchao mountain (33°41′N, 120°00′E), Hangzhou, China. The voucher specimen was deposited at Zhejiang A&F University, China under the accession no. JBCY001 (HuaChao Xu, xhcinsect@zafc.edu.cn). Genomic DNA was exacted from a larva using a DNeasy Blood & Tissue kit (QIAGEN, Hilden, Germany). Sequencing work of the complete mitogenome of Pyrocoelia analis was performed by Illumina Novaseq6000 in Shanghai Lingen Biological Technology Co., Ltd. (Shanghai, China), with a total data volume 5G (150 bp reads). The mitogenome was assembled by the SPAdes (Bankevich et al. 2012), and then was annotated using CGView. Maximum-likelihood (ML) trees were constructed using IQ-TREE (Trifinopoulos et al. 2016).

The total mitogenome length (GenBank accession no. OK323960) is 14,785 bp, including 13 typical invertebrate protein-coding genes (PCGs), 22 transfer RNA genes, and two ribosomal RNA genes. The A&T content of the whole *Pyrocoelia analis* mitogenome is 77.42% showing an obvious AT mutation bias (Eyre-Walker 1997).

All the 13 PCGs use standard ATN (Met) start codons (ATG in *ATP6*, *COX3*, *CytB*; ATT in *ND2*, *COX1*, *ATP8*; ATA in *COX2*, *ND6*, *ND3*; CAT in *ND4*, *ND4L*; AAT in *ND5*; TAT in *ND1*). The stop codons of the PCGs include four TAA (*ND2*, *ND6*, *CytB*, and *ATP8*), three TAG (*COX1*, *COX2*, and *ND3*), three TTA (*ND4*, *ND4L*, and *ND5*), and one CTA (*ND1*), whereas *ATP6* and *COX3* terminated with incomplete stop codon T (Yin et al. 2012). The most frequently used codons (ATT-Ile, TTA-Leu, TTT-Phe, ATA-Met, AAT-Asn, and TAT-Tyr) are composed of T or A + T, whereas the least used codons (ACG-Thr, CTG-Leu, GCG-Ala, CGC-Ser, etc.) have a high content of G + C. The relative synonymous codon usage (RSCU) exhibits extensive similarity with other Sciaridae mitogenomes in a previous study. Codon usage of PCGs shows a significant bias of high A + T content, which plays a major role in the A + T bias of the entire mitogenome (Yang et al. 2013). The 22 tRNA genes vary in size from 61 bp to 71 bp, whereas the 12S and 16S rRNA genes are 732 bp and 1247 bp in length, respectively.

Based on all PCGs of nine published Lampyridae and a Rhagophthalmidae mitogenomes, the phylogenetic tree (Figure 1) was constructed using ML and Bayesian inference (BI). The result showed that *Pyrocoelia analis* is closely related to *Pyrocoelia rufa*, which is in accordance with the traditional morphological classification. The mitogenome data from this study provide a better understanding for the phylogenetic relationship and speciation of Lampyridae.

**Figure 1.**
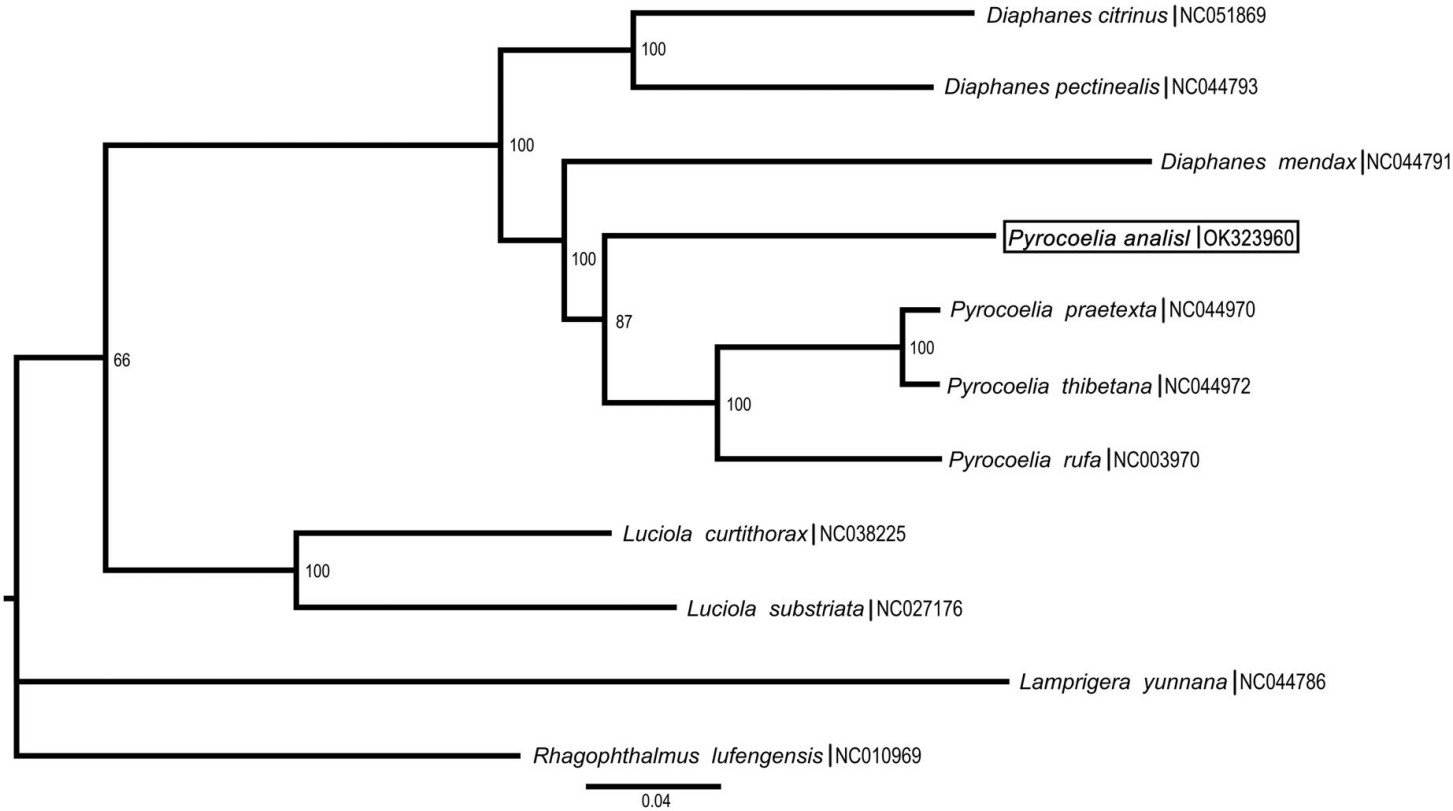
Phylogenetic tree of 11 of eight species of Lampyridae. The number on branches refers to the degree of confidence (%). Clades are labeled with names of subfamily or genus group of the family.

## Data Availability

The genome sequence data that support the findings of this study are openly available in GenBank of NCBI at https://www.ncbi.nlm.nih.gov/nuccore/OK202360.1/ under the accession no. OK323960. The associated BioProject, SRA, and Bio-Sample numbers are PRJNA765536, SRR16019617, and SAMN21566058, respectively.
